# Structural and functional analysis of somatic coding and UTR indels in breast and lung cancer genomes

**DOI:** 10.1038/s41598-021-00583-1

**Published:** 2021-10-27

**Authors:** Jing Chen, Jun-tao Guo

**Affiliations:** Department of Bioinformatics and Genomics, University of North Carolina at Charlotte, Charlotte, NC 28223 USA

**Keywords:** Genome informatics, Cancer genomics

## Abstract

Insertions and deletions (Indels) represent one of the major variation types in the human genome and have been implicated in diseases including cancer. To study the features of somatic indels in different cancer genomes, we investigated the indels from two large samples of cancer types: invasive breast carcinoma (BRCA) and lung adenocarcinoma (LUAD). Besides mapping somatic indels in both coding and untranslated regions (UTRs) from the cancer whole exome sequences, we investigated the overlap between these indels and transcription factor binding sites (TFBSs), the key elements for regulation of gene expression that have been found in both coding and non-coding sequences. Compared to the germline indels in healthy genomes, somatic indels contain more coding indels with higher than expected frame-shift (FS) indels in cancer genomes. LUAD has a higher ratio of deletions and higher coding and FS indel rates than BRCA. More importantly, these somatic indels in cancer genomes tend to locate in sequences with important functions, which can affect the core secondary structures of proteins and have a bigger overlap with predicted TFBSs in coding regions than the germline indels. The somatic CDS indels are also enriched in highly conserved nucleotides when compared with germline CDS indels.

## Introduction

Insertion and deletion (indel) is an important variation type in the human genome, second only to the single nucleotide variations (SNVs)^1–8^. Previous studies have estimated that indels contribute to 16% to 25% of sequence polymorphisms in human populations^2,9–11^. Like other types of variations, indels can alter human traits and lead to diseases including cancer^12–15^. Indel analyses have been carried out in both healthy and cancer genomes. In 2011, about 1.96 million small indels were identified in 79 human genomes, which was reported to have more than 97% validation rate^16^. The 1000 Genomes Project reported 1.48 million indels in 2010. There are 463,377 common indels between the above two studies, a result that reflects a combination of indel diversity and inaccurate indel annotations^1,16,17^. In addition to indels in healthy human genomes, efforts have been carried out to investigate indels in different cancer types. Recent pan-cancer analyses indicated the substantial variations among different cancer types^18–20^. Similar to indel annotation in heathy genomes, different methods and algorithms may lead to different somatic indel annotations^17^.

In coding regions, an indel can be frameshift (FS) or non-frameshift (NFS) depending on the length of an insertion or a deletion^2,16^. If the length of an indel is a multiple of three nucleotides, it is an NFS indel as it only affects the amino acid(s) of the indel while other coding indels that change the open reading frame are considered FS indels. For germline indels in healthy human genomes, the number of FS indels is much lower than expected, suggesting FS indels are potentially deleterious and less tolerated during evolution^21^. Several programs have been developed to predict the potential disease-causing NFS and FS indels^22–27^. To better understand the role of somatic indels in cancer genomes, studies have been done at both domain and protein level. Pagel et al*.* mapped somatic NFS indels from COSMIC onto protein structures and found that pathogenic variants tend to be enriched in helical and stand regions of protein structures^28^. Niu et al*.* developed a tool to identify 3-dimentional (3D) variants clusters on protein structures that can be used in variant-drug interaction analysis in cancer genomes^29^. Among the mutation-mutation and mutation-drug clusters from more than 4,400 samples across 19 cancer types, more than 6000 clusters were identified at 3D structure level, including both intra-molecular and inter-molecular clusters. They reported that about 0.76% of the 553,496 somatic variants are indels^29^.

Mutations in non-coding regions can also cause diseases^30–37^. Most analyses on non-coding variants in the regulatory regions in cancer genomes either focused on SNVs or did not differentiate SNVs from indels with relatively small sample sizes or a single cancer type^38,39^. Sakthikumar et al*.* investigated non-coding variants in Glioblastoma (GBM) genomes and demonstrated that the GBM somatic variants are enriched in non-coding regions of 78 GBM key genes^40^. Imielinski et al*.* showed that somatic non-coding indels in 79 lung adenocarcinoma genomes are exclusively enriched in surfactant protein genes^41^. Nakagomi et al*.* further analyzed 113 lung cancer samples and reported that other cancer types in lung also harbour non-coding indels and demonstrated the important role of those indels in lung cancer research^42^.

While eukaryotic genomes generally are considered to have two major types of sequences: (1) coding-sequences (CDSs) that encode proteins or RNAs, and (2) non-coding sequences that include regulatory regions such as promoters and enhancers for regulation of gene expression, a number of studies have shown that sequences in CDSs and the untranslated regions (UTRs) can also function as enhancers^43–46^. Mutations in coding and UTR enhancers can cause diseases by changing their enhancer activities^47–49^. Recent large-scale studies have shown that transcription factor binding sites (TFBSs) exist in coding regions in both human and mouse genomes based on ChIP-seq data analyses^47,50^. About 15% of codons in the human genome were hypersensitive to DNase I treatment, suggesting the existence of likely dual-use sequences for both amino acid coding and transcriptional regulation^50^. These dual function sequences, termed as duons, were considered to be more conserved than non-duon coding sequences and mutations in these duons could lead to diseases^50,51^.

The goal of this study is to investigate the potential role of somatic indels and the overlap between somatic indels and TFBSs in two of the most analyzed cancer types, invasive breast carcinoma (BRCA) and lung adenocarcinoma (LUAD). BRCA has the second largest proportion of indels among 19 cancer types^18,52^. LUAD has a high number of exonic somatic variants as reported in several studies^52,53^. Since the BRCA and LUAD sequences in TCGA data portal (https://portal.gdc.cancer.gov) are whole exome sequences, we focused our indel analysis on coding and the non-coding UTRs. In addition, while in principle the Whole Exome Sequencing (WXS) technology does not produce whole transcripts, studies have shown that 40–60% of the reads from exome sequencing are outside of the designed target regions including introns and these reads can be of high quality^54–57^. Therefore, besides the coding regions and UTRs, we also compared the indels in other regions of the transcripts as a side analysis.

Since somatic indel calling programs also predict germline indels found in healthy genomes^17^, we first identified these types of indels and removed them for downstream somatic indel analyses, including structural analysis of the effect of somatic NFS indels on protein secondary structure types, and gene enrichment and conservation analyses. We also mapped the somatic indels on the significantly mutated genes (SMGs) across major cancer types identified by Kandoth et al*.*^58^. More importantly, we investigated the somatic indels on the coding regions and UTRs that overlap with TFBSs, the dual-use sequences. To the best of our knowledge, this is the first large scale comparative study of mapping somatic coding indels to TFBSs.

## Materials and methods

### Sequence data and somatic indel calling

The 436 BRCA and 564 LUAD whole exome sequencing data, TCGA-BRCA and TCGA-LUAD, were downloaded from TCGA data portal at https://portal.gdc.cancer.gov (dbGaP Study Accession: phs000178.v11.p8). Both tumor and normal blood/tissue sequencing data were used to call somatic indels using the human genome reference GRCh38.p13 and Strelka^59^. Previous studies have demonstrated that Strelka performed well for somatic variants calling^60–62^. The indel set from the GATK Resource bundle with 1,267,008 germline indels was used as the reference of germline indel annotations in healthy human genomes (https://storage.cloud.google.com/genomics-public-data/resources/broad/hg38/v0/Mills_and_1000G_gold_standard.indels.hg38.vcf.gz). The transcript agreed by several references or the longest transcript for each gene was selected for annotating coding sequences and UTRs in both cancer exome sequences and germline sequences. In previous studies, a position *i* ± 5 has been used to determine whether two indels are the same, without concerning the indel types (insertion or deletion)^63^. Here we used a more stringent approach to identify the germline indels predicted as somatic indels by considering the indel types and insertion/deletion sequences in addition to the indel positions. Two indels are considered the same only if both have the same positions, indel types and sequences when comparing the predicted somatic cancer indels and the germline indels in the GATK Resource bundle. Since somatic indels are less conserved than the germline indels, we used the following two criteria: (1) the positions of two indels are within *i* ± 5; and (2) same insertion/deletion type to check the overlap of somatic indels between BRCA and LUAD cancer genomes.

### Protein secondary structure type analysis of coding indels

To locate the positions of coding somatic indels, we downloaded all protein coding gene annotations from Ensembl^64^. Each transcript with indel(s) was first searched against proteins with known structures in Protein Data Bank (PDB)^65^ using BLAST^66,67^. If a protein has a known structure or highly homologous protein structure (with at least 50% coverage and 80% sequence identity), the secondary structure types of the protein or the template protein were used. The protein secondary structure types of the protein were assigned with DSSP^68^. Of the eight secondary structure types from DSSP, H (α-helix), G (3_10_-helix) and I (π-helix) states were grouped as helix conformations; E (extended strand) and B (residue in isolated β-bridge) states were grouped as strand conformations and all the remaining states were considered as loop conformations^68^. If no known structures were found in PDB, RaptorX-Property was applied to predict secondary structure types with default settings^69^. RaptorX-Property uses conditional neural fields method to predict secondary structure types and achieves close to 84% Q3 prediction accuracy based on five different datasets^69^. The structural analysis of the germline indels from healthy genomes were performed with the 1370 coding indels annotated by the GATK Resource bundle.

### Overlap of indels with TFBSs

To investigate the overlap between somatic/germline indels and TFBSs, we used the TFBS set predicted with dePCRM2, a recently developed program for genome scale TFBS prediction with a high sensitivity of more than 97%^70^. A total of 25,297,119 non-overlapping TFBSs were predicted using dePCRM2 with a p-value cutoff of 5 × 10^–6^.

### Gene enrichment analysis and assignment of conservation scores

To investigate the functional categories of the genes affected by somatic coding indels in BRCA and LUAD, we applied DAVID 6.8 (the Database for Annotation, Visualization and Integrated Discovery) to perform functional enrichment analysis^71^. A cutoff of 0.001 was set for the adjusted p-values with Bonferroni correction to identify the significantly enriched terms in biological process or molecular function.

The phyloP scores of each nucleotide position in the human genome were downloaded from the UCSC Genome Browser database^72,73^. The two flanking nucleotides for each insertion site and the deletion sequences were collected for phyloP distribution analysis as well as for finding genes with high phyloP conservation scores.

## Results

### Comparison of somatic indels between BRCA and LUAD

We found 109,856 and 91,159 somatic indels from 436 BRCA samples and 564 LUAD samples respectively with 16,909 common indels between them (Table 1). As a reference, a total of 498,938 germline indels were mapped to transcripts in healthy genomes from the GATK Resource bundle. Since the predicted indels by somatic indel prediction algorithms include germline indels (false somatic indels), these germline indels need to be filtered out first for meaningful downstream analysis^17^. As described in the Materials and Methods section, two indels are considered the same only if both have the same positions, indel types and sequences when comparing the germline indels from the GATK Resource bundle and the predicted somatic indels from BRCA and LUAD. We found that 16.74% and 19.64% of indels in BRCA and LUAD respectively are the same as the germline indels (Table 1). After removing the germline and non-transcript indels, 61,543 and 43,684 somatic transcript indels for BRCA and LUAD respectively were used for further analysis. Not surprisingly, the overlapped indels between BRCA and LUAD have a higher percentage of germline indels (23.16%) since germline indels are more conserved than the somatic indels within populations of different cancer types^21^.

**Table 1:** Somatic transcript indels in BRCA and LUAD.

Cancer type	# of total indels	Overlap with germline indels	# of somatic transcript indels*	Deletions	Insertions	# of transcripts with indels
BRCA	109,856	18,391 (16.74%)	61,543	36,109 (58.67%)	25,434 (41.33%)	14,519
LUAD	91,159	17,900 (19.64%)	43,684	27,148 (62.15%)	16,536 (37.85%)	13,593
BRCA ∩ LUAD	16,909	3916 (23.16%)	9988	5330 (53.36%)	4658 (46.64%)	6600
Germline	1,267,008	–	498,938	284,597 (57.04%)	214,341 (42.96%)	17,278

*The numbers of somatic transcript indels in BRCA and LUAD are indels on transcripts after removing the ones that overlap with germline indels.

Similar to germline indels in healthy genomes, relatively more deletions than insertions were found in BRCA and LUAD. The percentages of deletions in both cancer types are slightly higher than those in the GATK germline indel set (Table 1). The distributions of insertion/deletion in both BRCA and LUAD are significantly different from germline indels (chi-squared test, p-value = 4.532 × 10^−16^ for BRCA and p-value < 2.2 × 10^−16^ for LUAD). The number of transcripts that have somatic indels are 14,519 and 13,593 in BRCA and LUAD respectively. It should be noted that while the whole exome sequences from the BRCA and LUAD contain all the coding and UTRs, they do not have the whole transcript sequences as the healthy genomes do. Therefore, at the transcript level, the somatic indels are undercounted.

### Somatic coding indels in BRCA and LUAD genomes

As shown in Table 2, the number of transcripts with somatic coding indels and the number of coding indels in both BRCA and LUAD are much higher than those of germline coding indels in healthy genomes (Table 2). The proportions of somatic coding indels are 8.64% in BRCA and 17.89% in LUAD while it is only 0.62% for the germline coding indels in healthy genomes. In terms of the deletion/insertion ratio in coding regions, LUAD has more deletion types (~ 70%) than that in the germline indels from healthy genomes (64.6%) while about 57.86% of somatic coding indels from BRCA samples are deletions. For the overlapping indels between BRCA and LUAD, the deletion and insertion are about 43.6% and 56.4% respectively.

**Table 2:** Somatic coding (CDS) indels in BRCA and LUAD.

Cancer type	# of transcripts with CDS indels	CDS indels*	Deletions	Insertions	FS indels	NFS indels
BRCA	3979	5320 (8.64%)	3078(57.86%)	2242 (42.14%)	3947 (74.19%)	1373(25.81%)
LUAD	5458	7813 (17.89%)	5526(70.73%)	2287 (29.27%)	6387 ( 81.75%)	1426(18.25%)
BRCA ∩ LUAD	798	835(8.36%)	364(43.59%)	471 (56.41%)	496 (59.40%)	339(40.60%)
Germline	1180	1370 (0.62%)	885(64.60%)	485 (35.40%)	679 (49.56%)	691(50.44%)

*The percentages are calculated against the number of transcript indels.

Coding indels are typically divided into FS and NFS types based on the length of indels. FS indels cause a reading frame shift at the indel site, which are prone to be more deleterious^21,74,75^. Our previous analysis of healthy genomes from the 1000 Genomes Project revealed that the number of germline FS indels is similar to that of NFS indels^21^. We also observed a similar pattern from germline coding indels in the GATK Resource bundle, 679 FS vs. 691 NFS (Table 2). These results indicate that healthy genomes tend to have much fewer FS coding indels than expected. However, for somatic coding indels in cancer genomes, the number of FS indels is about 2.8 (BRCA) to 4.5 (LUAD) times more than that of NFS indels. Over eighty percent of the somatic coding indels in LUAD are FS indels (Table 2). The overlapped coding somatic indels between BRCA and LUAD genomes have a relatively lower ratio of FS indels (59.4%), but it is still much higher than that in the germline (49.56%).

The somatic coding indels affect a total of 8,286 genes when BRCA and LUAD are combined. BRCA and LUAD have somatic coding indels in 3,979 and 5,458 genes respectively and 798 genes have somatic coding indels in both BRCA and LUAD. Among these genes, MAP3K1 has the most somatic coding indels in BRCA (45 indels), and TP53 has the most somatic coding indels in LUAD (37 indels) (Table 3). We compared the top genes with multiple somatic coding indels in our datasets to the 125 protein coding SMGs among the 127 total SMGs across 12 major cancer types (The other two are one lncRNA gene and one miRNA gene)^58^. Seven and five of the top 10 genes with multiple somatic coding indels in BRCA and LUAD respectively are in the list of 125 protein coding SMGs while none of the top 10 genes with germline coding indels are in the 125 SMGs (Table 3). Some of these genes have served as targets for drug development, such as EGFR^76^. Functional enrichment analysis revealed that the genes with somatic coding indels in LUAD are highly enriched in biological processes involved in cell adhesion while the indels in BRCA affect more chromatin remodelling and transcription (Supplementary Table S1).

**Table 3:** Top ten genes with multiple somatic CDS indels in BRCA and LUAD.

BRCA*	LUAD*	BRCA ∩ LUAD*	Germline
**MAP3K1**	**TP53**	**TP53**	SSPOP
**GATA3**	**STK11**	PABPC3	HLA-DRB1
**TP53**	TTN	MUC5B	TEKT4
**CDH1**	MUC16	HAVCR1	OR4C5
DSPP	**KEAP1**	PABPC1	SCYGR8
**KMT2C**	RBM10	ACIN1	MAML3
**PIK3R1**	RYR2	ZFHX4	ZFPM1
SPEN	CSMD3	**EPHB6**	ABCA10
**TBX3**	**NF1**	ZAN	KRT14
TTN	**EGFR**	FAM71D	MYO15B

*The genes are ranked by the number of somatic CDS indels and the genes in bold are the ones in the 125 SMG list.

**Figure 1:**
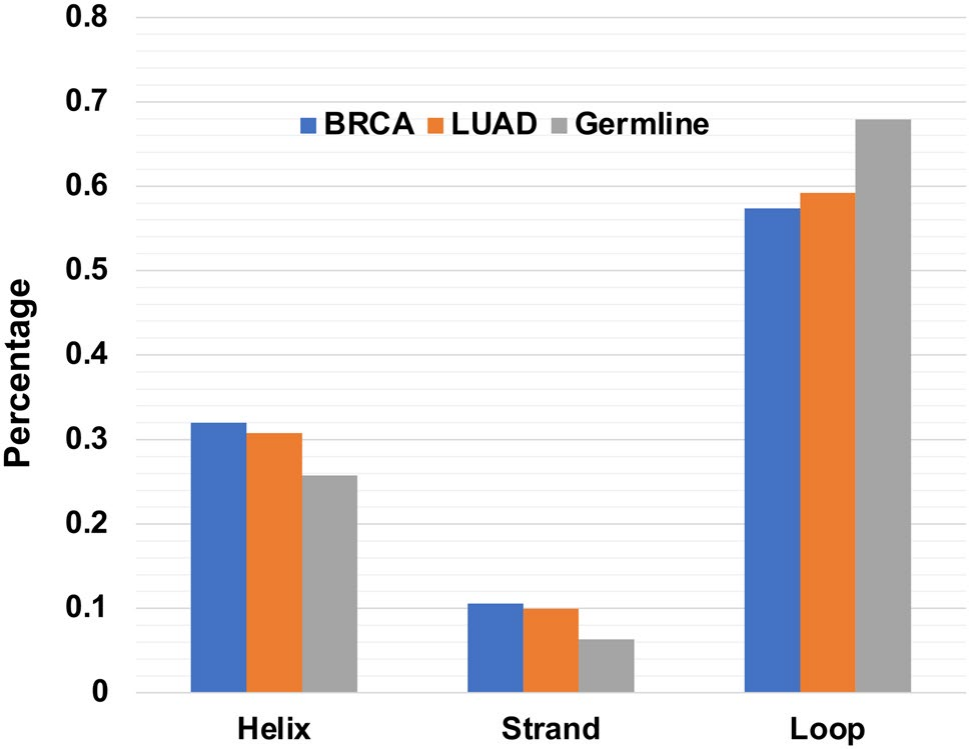
Distribution of secondary structure types of somatic NFS indels and germline NFS indels.

Since NFS somatic coding indels only affect part of the protein while keeping the remaining sequence unchanged, we compared the distributions of secondary structure types of these indels with the germline indels in healthy genomes. Among the proteins with NFS somatic coding indels, 181 and 106 proteins in BRCA and LUAD respectively were found to have known or homologous structures in PDB. For proteins having NFS somatic coding indels without known structures, we used RaptorX-Property to predict the secondary structure types for each amino acid of the indels, as described in the Materials and Methods section. While the distributions between the two cancer types are slightly different (chi-square test, p-value = 0.003), both are significantly different from that in germline indels (Fig. 1). Somatic coding NFS indels in cancer genomes have more helix and strand conformations with fewer loop types (chi-square test, p-values < 2.2 × 10^−16^), suggesting the NFS coding indels in both BRCA and LUAD cancer genomes affect more core secondary structures in the encoded proteins and are potentially more deleterious than the germline NFS coding indels in healthy genomes.

### Somatic non-coding UTR indels in BRCA and LUAD cancer genomes

For exonic non-coding somatic indels, we found 372 and 1940 indels in 5ʹ UTR and 3ʹ UTR respectively in BRCA, and 375 and 1187 indels in 5ʹ UTR and 3ʹ UTR respectively in LUAD (Supplementary Table S2). There are more somatic indels in 3ʹ UTR than those in 5’ UTR. In both BRCA and LUAD genomes, the indels are enriched in both 5ʹ UTR (0.66% and 1.06% for BRCA and LUAD, respectively) and 3ʹ UTR (3.45% and 3.31% for BRAC and LUAD, respectively) when compared with those in germline indels of healthy genomes, with 0.18% and 2.55% in the 5ʹ UTR and 3’UTR respectively (Supplementary Table S2). The majority of transcript somatic indels are located in the non-CDS, non-UTR regions. Therefore, even though the goal of whole exome sequencing is to get the exonic sequences, exome sequencing can generate high quality data and cover large non-target regions^54,55^. However, since the coverage of non-target regions in each cancer sample might be different from whole exome sequencing, it is difficult to draw conclusions when comparing the non-CDS, non-UTR noncoding transcript indels between two different cancer types and between cancer somatic indels and the germline indels.

### Conservation analysis of somatic CDS and UTR indels

**Figure 2:**
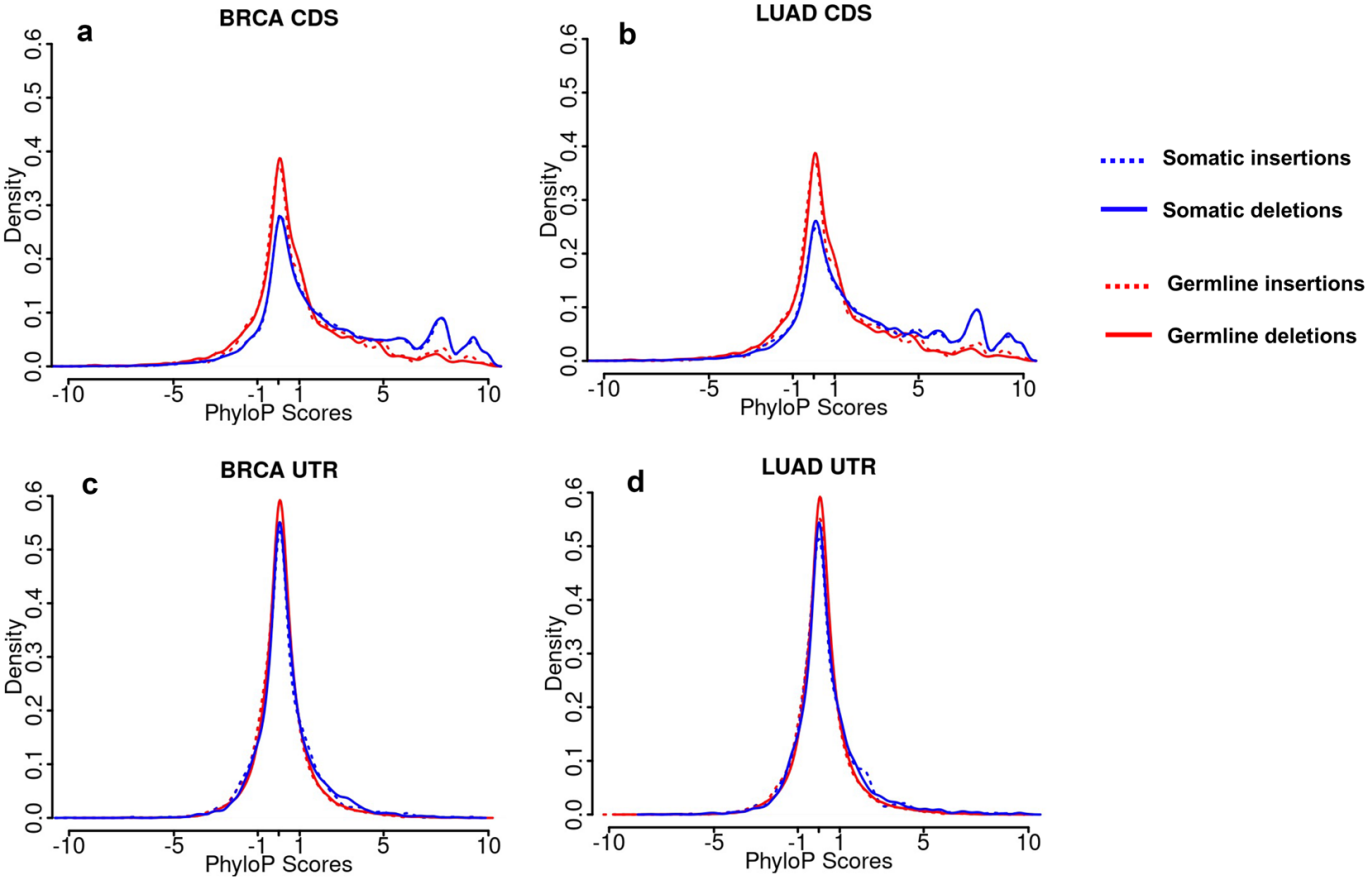
Distributions of the phyloP scores in somatic and germline CDS and UTR indels. (**a**) BRCA CDS indels; (**b**) LUAD CDS indels; (**c**) BRCA UTR indels; and (**d**) LUAD UTR indels. Blue is for somatic indels and red is for germline indels. The dashed line represents insertions and the solid line represents deletions.

It is interesting to see how conserved the somatic CDS and UTR indel sequences are in BRCA and LUAD when compared with germline CDS and UTR indels in healthy genomes. To this end, we compared the phyloP scores for nucleotides at the indel positions^72^. Since the phyloP scores of nucleotides are based on the reference genome, we collected the nucleotides for insertions and deletions differently. For insertion cases, the phyloP scores of the two flanking nucleotides at the indel site were collected while phyloP scores for the whole deletion sequences were considered (see Materials and Methods). The larger a positive phyloP score of a nucleotide position in the genome, the more conserved of the position. Figure 2 shows the distributions of phyloP scores of insertions and deletions in CDS regions (Fig. 2a,b) and UTR (Fig. 2c,d) in BRCA (Fig. 2a,c) and LUAD (Fig. 2b,d) respectively. In CDS regions, the somatic insertions and deletions have more positions with higher phyloP scores when compared with the distribution of germline insertions and deletions. There seems no apparent differences between BRCA and LUAD as well as between insertion and deletion cases. As for UTR indels, there is a difference between the cancer indels and germline indels. However, the differences are very small, especially when compared with those in the CDS positions.

### Overlap between somatic indels and TFBSs

The percent overlap between the somatic indels in cancer transcripts and TFBSs is larger than that between germline transcript indels and TFBSs (Table 4). The number of somatic CDS indels that overlap with TFBSs is much higher in cancer genomes, 2367 and 3140 in BRAC and LUAD respectively while there are only 520 for germline coding indels, suggesting cancer coding indels are enriched in these dual-functional regions. Somatic non-CDS transcript indels in cancer genomes are also enriched in the predicted TFBS sequences (25.4% and 27.01% for BRCA and LUAD, respectively) when compared to 17.41% in germline non-CDS transcript indels in healthy genomes (Table 4). A detailed look at these non-CDS transcript indels shows that there is a smaller percentage of overlap between 5ʹ UTR and TFBSs in BRCA and LUAD than that in healthy genomes while the 3’UTR is the opposite (Supplementary Table S3). While we also found that other non-CDS, non-UTR transcript indels have a larger percent overlap with TFBSs in cancer exomes, unlike the CDS cases, incomplete transcript sequences in the intron regions from exome sequencing makes it harder to make a fair comparison with the germline cases.

**Table 4:** Somatic transcript indels overlapping with TFBSs.

Cancer type	Transcript indels	Overlapping with TFBSs	CDS indels	CDS indels overlapping with TFBSs	Non-CDS transcript indels	Non-CDS indels overlapping with TFBSs
BRCA	61,543	16,646 (27.05%)	5320	2367 (44.49%)	56,223	14,279 (25.40%)
LUAD	43,684	12,830 (29.37%)	7813	3140 (40.19%)	35,871	9690 (27.01%)
BRCA ∩ LUAD	9988	2977 (29.81%)	835	332 (39.76%)	9153	2645 (28.90%)
Germline	498,938	87,156 (17.47%)	1370	520 (37.96%)	497,568	86,636 (17.41%)

We also performed conservation enrichment analysis with a phyloP score cutoff of 5 for indel positions in CDS with TFBS overlap and indels positions in CDS without TFBS overlap. The genes were ranked by the number of indel positions with phyloP scores above the cutoff in each case. The top 10 genes in each case are listed in Table 5. More SMG genes were found in indels with CDS and TFBS overlap than those CDS indels without TFBS overlap in both cancer types (7 vs. 4 in BRAC and 4 vs. 1 in LUAD) (Table 5). Not surprisingly, none of the 125 SMG protein coding genes were found in the germline indels no matter if CDS overlaps TFBS or not.

**Table 5:** Top ten genes with multiple high phyloP scores (> 5) in somatic CDS indels.

BRCA		LUAD		Germline	
CDS overlap with TFBS	CDS not overlap with TFBS	CDS overlap with TFBS	CDS not overlap with TFBS	CDS overlap with TFBS	CDS not overlap with TFBS
**GATA3**	**PIK3CA**	**STK11**	ADGRL3	LZTR1	RFX7
**MAP3K1**	**PIK3R1**	**TP53**	CDH8	ZEB2	CLTCL1
**TP53**	**MAP3K1**	**EGFR**	LRFN5	CDK8	RBBP6
**PTEN**	**PTEN**	TAF2	ATP2B1	GJB7	CHD9
YTHDF2	TTN	**APC**	**RPL5**	DBX1	NEDD4
**CDH1**	TM9SF2	PCDH9	KCNH7	GMNC	RERE
TM9SF4	PLCE1	MEAF6	CNOT1	OR5AU1	CARD11
**TBX3**	EPHB3	TUBB8B	DHX9	SRRM3	HYDIN
TTN	ADCYAP1R1	DOCK5	PSEN2	ZNF730	TMCC1
**RUNX1**	PDE11A	TAPT1	COG2	SPON1	DNAJC28

*The genes in bold are among the125 SMG list.

### Somatic indels in SMGs

We mapped the somatic indels to the annotated 125 protein coding SMGs and found that somatic indels in cancer genomes are enriched in SMGs, especially they are enriched in SMG’s coding regions in both BRCA and LUAD cancer genomes when compared with the germline indels from healthy genomes (Table 6). In healthy genomes, there are only 11 (0.23%) SMG indels in coding regions, but in BRCA and LUAD cancer genomes, 349 (33.82%) and 267 (38.98%) of SMG somatic indels are located in the coding regions respectively (Table 6). Among the 125 SMGs, 70 and 71 of them have BRCA and LUAD somatic indels in CDS regions respectively while only 9 SMGs have germline coding indels. Twelve SMGs have somatic coding indels in both cancer types, suggesting different mutation/variant patterns in different cancer types while there are some commonalities between cancer types.

**Table 6:** Somatic transcript indels in 125 SMGs of BRCA and LUAD.

Cancer type	Indels in SMGs	SMGs with indels in CDS regions	Indels in SMGs’ CDS	SMG CDS indels overlapping with TFBSs	Indels in SMGs’ non-CDS regions	SMG non-CDS indels overlap with TFBSs
BRCA	1032	70 (56.00%)	349 (33.82%)	172 (49.28%)	683	154 (22.55%)
LUAD	685	71 (56.80%)	267 (38.98%)	132 (49.44%)	418	105 (25.12%)
BRCA ∩ LUAD	129	12 (9.6%)	19 (14.73%)	9 (47.37%)	110	35 (31.82%)
Germline	4818	9 (7.2%)	11 (0.23%)	5 (45.45%)	4807	620 (12.90%)

The overlap between SMG somatic coding indels with TFBSs is significantly more in BRCA and LUAD than that in germline indels in healthy genomes (Table 6). There are 172 (49.28%) and 132 (49.44%) somatic coding indels overlap with TFBSs in SMGs in BRCA and LUAD while there are only 5 (45.45%) such cases in healthy genomes (Table 6). The overlap between the non-CDS somatic indels and TFBSs in BRCA (22.55%) and LUAD (25.12%) is higher than that in healthy genomes (12.9%) as well.

## Discussion

With the advancement of biotechnology, especially the NGS technology, a large number of genomes have been sequenced for a variety of cancer types. Somatic variations in cancer genomes have been one of the main focuses in cancer studies, including variants in both coding and non-coding regions^77^. However, most of the studies in cancer genomes focused on SNVs^29,40,52^. In this study, we carried out a comparative study of the somatic indels in two major cancer types, BRCA and LUAD with their whole exome sequences and compared some of the features with germline indels from healthy genomes.

There are several novel aspects from this study. First, we removed the germline indels predicted from the somatic indels calling program before performing downstream analyses. We demonstrated previously that some of the predicted somatic indels are exactly the same as the germline indels in healthy human genomes^17^. Therefore, these indels are considered as false somatic indels and represent “noise” when analysing features in cancer genomes, which need to be filtered out. Secondly, we investigated the overlap between cancer somatic indels, especially the coding indels with TFBSs. Previous case studies as well as large-scale analyses revealed the existence of the so-called duons that encode amino acids and also serve as TFBSs^43–47,50^. The percentage of such DNA sequences with dual functions varies in species and by different TFBSs annotations. Based on ChIP-seq data, Birnbaym et al*.* showed that there are 7% and 6% of binding peaks located in protein coding regions in human genomes and mouse genomes respectively^47^. Using DNase I footprinting method, Stergachis et al*.* found that at least 14% of coding regions in human genomes can bind transcription factors^50^. The prevalence of such sequences and their implication in diseases suggest their important roles in human genomes and diseases^47–49,51^. Third, we compared the conservation score distributions of CDS and UTR indels between cancer genomes and healthy genomes. Finally, we assessed the structural effects of the coding somatic NFS indels and investigated somatic indels in the 125 SMGs identified from different cancer types.

The somatic indels from different cancer types vary greatly. As shown in Table 1, only 9988 somatic indels appear in both cancer types, which account for 16.23% of BRCA and 22.86% of LUAD somatic indels respectively. The somatic transcript indels in two cancer types have different proportion of indel types. LUAD has more deletions, more indels in coding regions and more FS indels, than the BRCA cancer type. In our datasets, we did not find any complex indels, which are formed by simultaneously deleting and inserting DNA fragments of different sizes at a common genomic location^78^. Our data on somatic coding indels revealed a number of top SMGs with most indel variations in BRCA and LUAD. Except for TP53, other top 10 mutated genes are different between BRAC and LUAD, suggestion involvement of different gene mutations in different cancer types (Table 3). Functional enrichment analysis also shows different biological processes involved in each type of cancer (Tables 1, 2, Supplementary Table S1).

Compared with germline transcript indels in healthy genomes, somatic transcript indels in cancer genomes have higher proportions involved in the CDS regions. Coding somatic indels also have a higher rate of FS types, especially in SMGs (Tables 2, 6). This phenomenon is not surprising since FS indels are prone to be deleterious^21,74,75^. More importantly, the somatic coding indels are more likely to be enriched in the structurally and functionally important regions of proteins than the germline indels in the heathy genomes. First of all, we found that the NFS somatic indels in BRCA and LUAD are enriched in helical and strand secondary structure types (Fig. 1). Helices and strands represent the core of protein structures. Changes in the core would more likely affect the stability of the protein and disrupt the structure, which in turn affect the function of the protein. Secondly, the somatic coding indels are enriched in coding regions that are also predicted as TFBSs, or duons (Tables 4, 6). Therefore, these indels not only affect the protein sequences, they can also change the regulation of gene expression. In addition, compared to germline indels, somatic CDS indels are enriched in positions that have high conservation score based on phyloP analyses, suggesting these indels are more deleterious (Fig. 2).

While the cancer whole exome sequencing data have all the coding and UTR sequences that can be compared directly with the germline coding and UTR sequences in healthy genomes, one of the limitations of the whole exome sequences is that they only have partial non-coding sequences for the transcripts. It would be interesting to see the differences in the non-coding regions among different cancer types and between germline indels and cancer somatic indels from a large-scale comparative analysis. More detailed analyses on structural and functional effect can be carried out in the future to investigate the structural basis for better understanding these somatic indels as previous work done on point mutations^79–82^ and if a somatic indel is deleterious^27^.

## Supplementary Information

Supplementary Information.

## Data Availability

The data used in this study were downloaded from the TCGA data portal at https://portal.gdc.cancer.gov (dbGaP Study Accession: phs000178.v11.p8).
