# Supplementary material for: Structural and functional analysis of somatic coding and UTR indels in breast and lung cancer genomes
Source: Sci Rep. 2021 Oct 27;11:21178. doi: 10.1038/s41598-021-00583-1 (PMC8551294; doi:10.1038/s41598-021-00583-1)
Supplement: Supplementary file 1 — Supplementary Information. [file 41598_2021_583_MOESM1_ESM.pdf]

Supplementary Information

Structural and functional analysis of somatic coding and UTR indels in breast and lung cancer genomes

Jing Chen and Jun-tao Guo*

Table S1. Functional enrichment analysis for genes with CDS indels

A: LUAD

Biological Process		Molecular Function	
homophilic cell adhesion via plasma membrane adhesion molecules	9.50E-12	calcium ion binding	5.20E-06
cell adhesion	5.50E-09	ATP binding	3.50E-05
detection of chemical stimulus involved in sensory perception of smell	2.90E-03	RNA polymerase II core promoter proximal region sequence-specific DNA binding	2.90E-04
membrane depolarization during action potential	2.90E-03	metal ion binding	2.90E-04
extracellular matrix organization	3.20E-03	chromatin binding	2.90E-04
regulation of ion transmembrane transport	8.40E-03	G-protein coupled receptor activity	9.30E-04
synapse assembly	8.40E-03	olfactory receptor activity	9.40E-04
transcription, DNA-templated	8.40E-03	extracellular-glutamate-gated ion channel activity	3.40E-03
axon guidance	8.40E-03	extracellular matrix structural constituent	3.40E-03
		ionotropic glutamate receptor activity	7.60E-03

B: BRCA

Biological Process		Molecular Function	
chromatin remodeling	2.30E-03	chromatin binding	4.60E-09
transcription, DNA-templated	2.70E-03	DNA binding	3.70E-04
		ATPase activity	5.40E-04
		transcription regulatory region DNA binding	3.30E-03
		actin binding	3.30E-03
		calcium ion binding	5.70E-03

Table S2: Somatic non-CDS transcript indels in BRCA and LUAD

Cancer type	# of non-CDS transcript indels	Indels in 5'UTR	Indels in 3'UTR	Indels in other non-CDS transcript regions
BRCA	56,223	372 (0.66%)	1,940 (3.45%)	53,912 (95.89%)
LUAD	35,871	375 (1.06%)	1,187 (3.31%)	34,309 (95.65%)
BRCA ∩ LUAD	9,153	89 (0.97%)	345 (3.76%)	8,719 (95.26%)
Germline	497,568	879 (0.18%)	12,674 (2.55%)	484,016 (97.28%)

Table S3: Somatic non-CDS transcript indels overlapping with TFBSs

Cancer type	Indels in 5'UTR	5'UTR indels overlapping with TFBSs	Indels in 3'UTR	3'UTR indels overlapping with TFBSs	Indels in other non-CDS transcript regions	Other non-CDS transcript indels overlapping with TFBSs
BRCA	372	175 (47.04%)	1,940	713 (36.75%)	53,912	13,391 (24.84%)
LUAD	375	184 (49.07%)	1,187	465 (39.17%)	34,309	9,041 (26.35%)
BRCA ∩ LUAD	89	41 (46.07%)	345	134 (38.84%)	8,719	2,470 (28.33%)
Germline	879	463 (52.67%)	12,674	4,164 (32.85%)	484,016	82,009 (16.94%)
